# Different Plaque Composition and Progression in Patients with Stable and Unstable Coronary Syndromes Evaluated by Cardiac CT

**DOI:** 10.1155/2015/401357

**Published:** 2015-08-03

**Authors:** Maiken Glud Dalager, Morten Bøttcher, Jesper Thygesen, Gratien Andersen, Hans Erik Bøtker

**Affiliations:** ^1^Department of Cardiology, Aarhus University Hospital, Palle Juul-Jensens Boulevard 99, 8200 Aarhus N, Denmark; ^2^Cardiac Imaging Center, Hospital Unit Vest, Gl. Landevej 61, 7400 Herning, Denmark; ^3^Department of Biomedical Engineering, Aarhus University Hospital, Palle Juul-Jensens Boulevard 99, 8200 Aarhus N, Denmark; ^4^Department of Radiology, Aarhus University Hospital, Palle Juul-Jensens Boulevard 99, 8200 Aarhus N, Denmark

## Abstract

*Objective.* To compare the quantity, subtype, and progression of atherosclerosis by cardiac computed tomography (CT) and intravascular ultrasound (IVUS) in patients with stable (SAP) and unstable angina pectoris or non-ST-elevation myocardial infarction (UAP/n-STEMI).* Methods.* Forty patients with SAP and 20 with UAP/n-STEMI underwent cardiac CT and angiography with IVUS at baseline and after one year. Atherosclerotic segments were divided into calcified, mixed, or noncalcified subtypes, and significant stenoses were registered.* Results.* Thirty-two SAP and 15 UAP/n-STEMI patients completed the CT follow-up. At baseline, the number of atherosclerotic segments was higher in UAP/n-STEMI than in SAP (*P* = 0.039). UAP/n-STEMI patients had more segments with noncalcified plaques (*P* = 0.0005) whereas SAP patients had more segments with calcified plaques (*P* = 0.013). The number of segments with significant stenosis did not differ between the groups, but noncalcified plaques more frequently caused significant stenoses in UAP/n-STEMI than in SAP patients (*P* = 0.0002). After one year the number of segments with atherosclerosis increased in SAP patients (*P* = 0.0001). The number of atherosclerotic segments remained unchanged in UAP/n-STEMI patients. However, composition was altered as the number of segments with noncalcified plaques decreased (*P* = 0.018). IVUS data confirmed the CT findings.* Conclusion.* Quantity, subtype, and progression of atherosclerosis differ between SAP and UAP/n-STEMI patients.

## 1. Introduction

Atherosclerotic plaques in the coronary arteries may lead to stenoses and stable angina pectoris while others after years of indolent growth suddenly undergo transformation to a vulnerable stage complicated by rupture and thrombus formation [1]. The mechanisms behind this development are largely unknown [2]. Autopsy studies indicate that plaque composition in patients presenting with stable angina pectoris (SAP), unstable angina pectoris (UAP), or myocardial infarction differs pathoanatomically [3–7]. Vulnerable or rupture prone plaques are characterized by a large lipid-rich core, a thin fibrous cap containing few smooth muscle cells and many macrophages, angiogenesis, adventitial inflammation, and outward remodeling [8–10].

It has been suggested that cardiac CT scanning enables characterization of differences in plaque composition in SAP and UAP and this has led to expectations about potential prediction of plaque rupture. Multidetector computed tomography (MDCT) can identify and characterize atherosclerotic plaques in the coronary arteries [11–13], and several studies have demonstrated associations between the presence of coronary artery disease on a CT scan and future cardiovascular events [14, 15]. However, the importance of plaque composition determined by MDCT in patients presenting with SAP or UAP/non-ST-elevation myocardial infarction (n-STEMI) remains unknown, as does the development of plaque composition, which may determine the clinical outcome in both disease entities.

The aim of the present prospective study was to compare the number and composition of atherosclerotic segments in patients presenting with SAP or UAP/n-STEMI and to assess differences in the progression of plaque composition after one year of follow-up.

## 2. Methods

### 2.1. Patient Population

From December 2008 to February 2010 patients referred for elective coronary angiography (CAG) at our center due to SAP defined as a history with physical exertion causing characteristic chest pain and fulfilling the additional inclusion criteria were invited by letter to participate in the study. Referral for elective CAG was decided independently by two experienced cardiologists and based on the European Society of Cardiology criteria for an a priori high-risk profile and typical angina symptoms [16]. One hundred and fifty-two patients were invited. Of these 40 accepted to participate in the study. Twenty patients with UAP/n-STEMI were recruited for the study upon admission to our department for a subacute CAG. Additional inclusion criteria were age >18 and <80 and sinus rhythm with a heart rate <100 beats per minute. UAP/n-STEMI patients were required to present with characteristic chest pain >15 min combined with significant ST-segment fluctuation (UAP) or significant rise and fall in biochemical ischemic markers (troponin T or I and creatine kinase-MB) (n-STEMI), in accordance with the guidelines from the European Society of Cardiology for the management of acute coronary syndromes in patients presenting without persistent ST-segment elevation [17].

Exclusion criteria were renal dysfunction with serum creatinine >100 *μ*mol/L, known allergy to contrast media, previous bypass operation, unstable clinical condition, severe heart failure, severe pulmonary disease or other contraindications to *β*-blockers, inability to perform a 10-second breath hold, pregnancy, and inability to give informed consent.

The local Ethics Committee approved the research protocol, and all patients gave informed consent.

### 2.2. Coronary CT Angiography

All patients underwent coronary CT angiography prior to the planned CAG at baseline, and again at follow-up for the patients completing the second examinations.

The coronary CT angiography (CCTA) was obtained using a 64-slice multidetector CT scanner (Brilliance 64, Philips Healthcare, Ohio, USA) or a Siemens Definition Flash (Siemens Health Care, Erlangen, Germany). We performed helical, retrospective ECG gated CT scans and used ECG pulsing of the R-R interval with tube current modulation of the retrospectively ECG gated acquisitions. As contrast medium Iomeron 350 mgI/mL was used in volumes of 75–90 mL, 5 mL/s depending on patient size. A 30 mL saline chaser was administered after contrast injection. The scanner settings for the Philips scanner were 120 kV, 381–500 mA depending on the patient size, gantry rotation speed of 0.4 s, pitch 0.2, and a detector collimation of 64*∗*0.625 mm. Minimal slice thickness was 0.9 mm and slices were reconstructed with 50% overlap. Images were reconstructed using a dedicated cardiac filter (CC-filter). Reconstructions were performed routinely at 40%, 75%, and 80% phases of the R-R interval period. The scanner settings for the Siemens scanner were 100–120 kV, 265–785 mA depending on the patient size, gantry rotation speed of 0.28 s, pitch 0.23, and a detector collimation of 2*∗*64*∗*0.6 mm with a flying focus spot. Slice thickness was 0.75 mm and slices were reconstructed with 20% overlap. Images were reconstructed using dedicated cardiac filters (B26f and I26f).

Since radiation dose was restricted, we did not perform noncontrast scans. Patients with a heart rate above 70 bpm received oral beta-blockers (25–50 mg of atenolol depending on the heart rate and blood pressure) one hour before the scans, and if necessary additional intravenous beta-blockers were given in the scanner-room just before the scan. Patients were given instructions regarding breath hold during preparation one hour before the scan and again in the scanner-room. All follow-up scans except one were performed on the same CT scanner as the inclusion scans and identical scanner settings were applied.

### 2.3. Coronary Angiography with Intravascular Ultrasound

After the CT scan all patients underwent CAG supplemented with intravascular ultrasound (IVUS). IVUS data were acquired using a phased-array, 20 MHz, 3.2-F IVUS catheter (Eagle Eye, Volcano Corporation, Rancho Cordova, CA). Motorized pull-back was performed with a pull-back rate of 0.5 mm/s. IVUS was intended in all three vessels and performed in one to three vessels depending on feasibility. Failure to obtain IVUS in a vessel was due to vessel anatomy, severe calcification, and patient related factors.

### 2.4. Image Analysis, CT

Images were analyzed using Philips Extended Brilliance Workspace, Philips Healthcare V4.0.1.162. The axial images were evaluated and reconstructed using multiplanar reconstruction. The window centre and width were fixed at 200 HU and 1000 HU, respectively.

The coronary tree was divided into a 17-segment model [13] based on the model suggested by AHA [18]. Segments 4, 8, 10, 12, 14, 15, 16, and 17 were considered of insufficient and variable size for routine examination. The remaining nine segments (1, 2, 3, 5, 6, 7, 9, 11, and 13) were analyzed and categorized as follows: segments not accessible, segments without plaque, segments with insignificant plaques, and segments containing significant plaques. A plaque was considered significant if it comprised more than 50% of the coronary artery diameter (corresponding to >70% of the coronary artery luminal area). The segments with plaques were further divided into calcified, noncalcified, or mixed subtypes depending on the primary plaque component. A plaque was considered calcified if it contained more than 50% calcifications, noncalcified if it contained less than 5% calcification, and mixed with calcification content between 5 and 50%.

The atherosclerotic plaques were identified as structures adjacent to or compromising the coronary artery lumen and clearly distinguishable from the surrounding tissue. Noncalcified plaques had lower density than the contrast-enhanced vessel lumen and appeared as darker structures. Calcified plaques had higher density than the contrast filled lumen and appeared as brighter structures. Finally mixed plaques were identified as structures containing both calcified and noncalcified components. Areas with stents at baseline were not evaluated. Segments stented at baseline were assigned to the same plaque-category at follow-up.

Two experienced readers, blinded to the patient category, analyzed the segments. In cases of disagreement, the segments were reevaluated until consensus was reached. Segments were considered to harbor a significant atherosclerotic plaque when the artery lumen was not clearly distinguishable, or the plaque gave rise to a stenosis comprising >50% of the coronary artery diameter. Both readers were blinded to the baseline results when scoring the follow-up scans.

### 2.5. Image Analysis, IVUS

To avoid bias one vessel was analyzed in each patient. The vessels were selected by the availability of IVUS at both baseline and follow-up. In case more than one vessel fulfilled this criterion, vessels were prioritized in the following order: LAD-RCA-Cx. As visual interpretation of the gray scale IVUS images is limited, we used radiofrequency data to establish virtual histology data for thorough plaque classification according to commercial analysis software (Volcano Image Analysis Software version: 3.0.394, Volcano Corp.). We determined areas and volumes of the four characteristic plaque components (fibrous tissue (green), fibrofatty tissue (yellow), necrotic core (red), and dense calcium (white)).

### 2.6. Statistical Analysis

Patient characteristics were compared using students *t*-test for continuous variables (age, ejection fraction, and body mass index (BMI)) and Chi-square/Fischer's exact test for categorical data. Normality was checked by histograms and qq-plots. As the number of segments with disease was not normally distributed, the comparison between the number and subtype of coronary artery segments with atherosclerosis in the two patients groups was performed by linear regression using robust variance estimates. Paired *t*-test was applied to test whether there was a statistical significant development in the amount and subtype of atherosclerosis after one year. Normality was confirmed by qq-plots and histograms of the differences and independency was checked by Bland-Altman plot. Results are reported as means ± 95% confidence interval (CI).

Our sample size calculations were based on the assumptions that the mean number of segments with atherosclerosis in the SAP group was “*n*” and the mean number in the UAP/n-STEMI was “*n* + 1.” Standard deviations within each group were estimated to be one, the power was 90%, and alpha was 5%. Furthermore we assumed that atherosclerosis in the UAP/n-STEMI group was more aggressive, leading to an increase in the number of segments with disease from “*n*” at baseline to “*n* + 1” at follow-up. We expected that the development of atherosclerosis in the SAP group would be slower than the development of atherosclerosis in the UAP/n-STEMI group, and we assumed that the number of segments with disease would increase from “*n*” at baseline to “*n* + 0.75” at follow-up.

These assumptions required a sample size of 22 patients in the UAP/n-STEMI group and 38 patients in the SAP group. A power of 80% required a sample size of 16 patients in the UAP/n-STEMI group and 28 patients in the SAP group. This number was reached in the investigation.

## 3. Results

### 3.1. Patient Characteristics

We included 40 patients with SAP and 20 patients with UAP/n-STEMI (two patients presented with UAP, and 18 patients presented with n-STEMI). All patients with SAP presented with chest pain. The patients with SAP were classified according to the Classification of angina severity according to the Canadian Cardiovascular Society, the CCS-classification. This classification scheme divides patients into four groups: Group 1: ordinary activity which does not cause angina such as walking and climbing stairs; Group 2: slight limitation of ordinary activity; Group 3: marked limitation of ordinary physical activity; Group 4: inability to carry on any physical activity without discomfort [16]. According to this classification eight patients were CCS-class 1, 23 patients were CCS-class 2, and nine patients were CCA-class 3. The patient characteristics at baseline are shown in Table 1. Age, BMI, smoking habits, ejection fraction, and gender-distribution did not differ between groups. Elevated blood pressure, dyslipidemia, and a family history of ischemic heart disease were significantly more prevalent in the SAP group than in the UAP/n-STEMI group, whereas type 2 diabetes mellitus was more prevalent in the UAP/n-STEMI group than in the SAP group.

Thirty-two SAP and 15 UAP/n-STEMI patients completed the one-year follow-up CT scan. Failure to complete follow-up was comparable in the two groups: referral to coronary bypass operation (*n* = 4 (SAP) and *n* = 3 (UAP/n-STEMI)), elevated creatinine levels (*n* = 2 (SAP)), and withdrawal of consent (*n* = 2 (SAP) and *n* = 2 (UAP/n-STEMI)). There was no difference in the proportion of patients, who completed follow-up: 32 out of 40 patients with SAP and 15 out of 20 patients with UAP completed the follow-up CT scan (*P* = 0.45). One patient with SAP was reexamined after only six months due to recurrence of symptoms. The CAG showed a new insignificant stenosis (40%) in the left main coronary artery; percutaneous coronary intervention (PCI) was not performed. The mean follow-up time for the remaining 46 patients was 355 (±12) days. At follow-up six patients needed additional PCI (five were SAP patients and one was a UAP/n-STEMI patient).

We achieved complete follow-up IVUS data in 22 SAP patients and ten UAP/n-STEMI patients.

Table 2 shows the medical treatment and smoking habits at follow-up. At baseline all patients were assigned to a comprehensive and similar cardiac rehabilitation program including initiation of relevant medical therapy, physical exercise, and enrollment in a smoking cessation program when appropriate. Therefore the follow-up data for risk factor modification reflects treatment throughout the study period. Almost all patients received aspirin and statins. More patients in the UAP/n-STEMI group than in the SAP group received clopidogrel. This was expected because clopidogrel was recommended for all patients with n-STEMI, regardless of the need for PCI. There was no difference in treatment with beta-blockers and ACE-inhibitors/AT2-antagonists between the two groups. During follow-up four patients in the UAP/n-STEMI group and two patients in the SAP group stopped smoking. There was still no difference in smoking habits between the groups. Information regarding compliance and medication during follow-up was obtained by questionnaires and hospital records.

### 3.2. Plaque Characteristics at Baseline, CT

A total of 462 out of 540 segments were analyzed; 78 segments were not analyzable due to the following: no separate left main artery (*n* = 7), occluded segments (*n* = 17), small or unidentified segments (*n* = 46; 42 of these were segments 9 and 13), and movement artifacts (*n* = 8). In the 462 segments 136 (29,4%) did not have atherosclerosis, 241 (52,2%) had atherosclerotic lesions, but no significant stenoses, and 85 (18,4%) segments had one or more significant stenoses.

**Figure 1:**
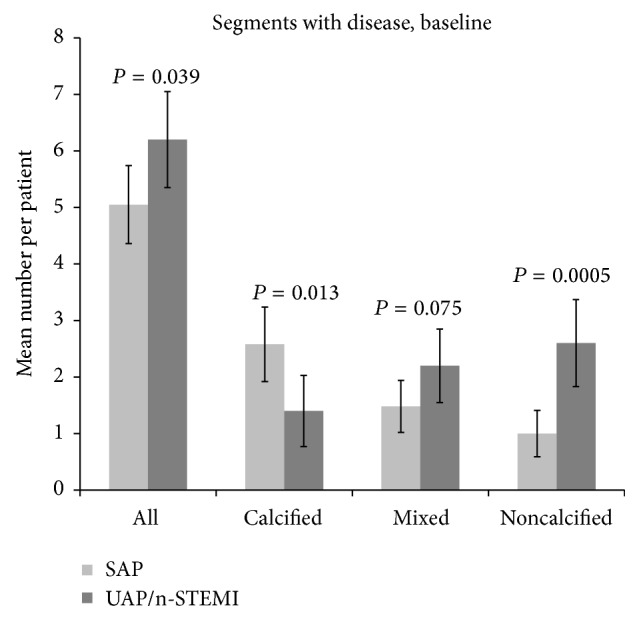
Mean number of segments with atherosclerosis in patients with stable angina pectoris (SAP) and patients with unstable angina pectoris/non-ST-elevation myocardial infarction (UAP/n-STEMI) at baseline. The first two rows display all segments with atherosclerotic plaques; the next six rows display the allocation of the segments into the three atherosclerotic subtypes.

The distribution of segments with disease between the two patient groups is displayed in Figure 1. The number of coronary artery segments with atherosclerotic disease was higher in UAP/n-STEMI patients than in SAP patients (6.2 ± 0.85 versus 5.1 ± 0.69, *P* = 0.039). More segments with noncalcified plaques were found in the UAP group than in the SAP group (2.6 ± 0.77 versus 1.0 ± 0.41, *P* = 0.0005), while more segments containing calcified plaques were found in the SAP compared to the UAP/n-STEMI group (2.6 ± 0.66 versus 1.4 ± 0.63, *P* = 0.013). There was no difference in the number of segments with mixed plaques between the two patient groups, despite a tendency towards more segments with mixed plaques in the UAP/n-STEMI group (2.2 ± 0.65 versus 1.5 ± 0.46, *P* = 0.075).

**Figure 2:**
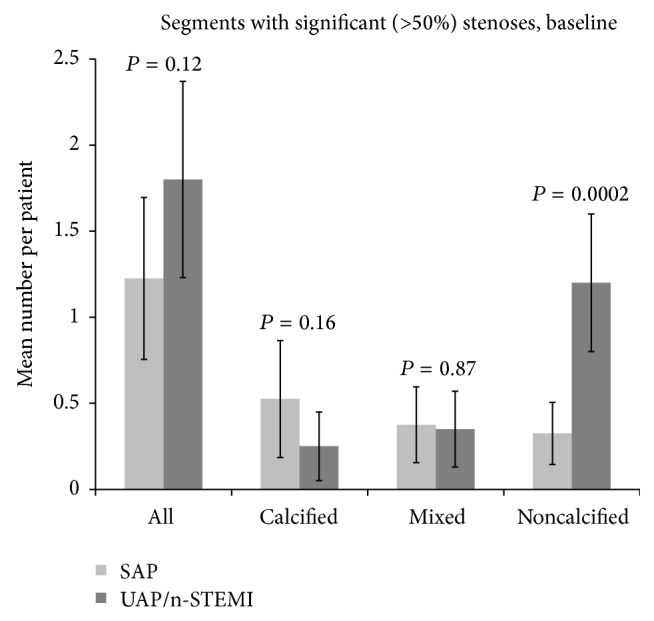
Mean number of segments with significant atherosclerotic plaques (>50% stenosis) in patients with stable angina pectoris (SAP) and patients with unstable angina pectoris/non-ST-elevation myocardial infarction (UAP/n-STEMI) at baseline. The first two rows display all segments with atherosclerotic plaques; the next six rows display the allocation of the segments into the three atherosclerotic subtypes.

Figure 2 shows the distribution of segments containing significant stenosis (>50% stenosis) in the two patient groups. Although there was no difference in the number of segments with significant stenosis between the groups (SAP 1.2 ± 0.47 versus UAP/n-STEMI 1.8 ± 0.57, *P* = 0.12), we identified more segments with significant stenosis caused by noncalcified plaques in the UAP/n-STEMI group compared with the SAP group (1.2 ± 0.4 versus 0.33 ± 0.18, *P* = 0.0002). We found no significant differences in the number of segments with significant stenosis caused by calcified or mixed plaques between the two groups.

The radiation dose for the CT scans ranged between 5 and 18.1 mSv.

### 3.3. Comparison of CT with IVUS and CAG at Baseline

Baseline IVUS data was achieved in 29 SAP patients and 19 UAP patients. Failure to obtain IVUS was due to hardware failure *n* = 5, IVUS impossible to perform due to severe three-vessel disease *n* = 3, and software problems *n* = 4. The IVUS data (Table 3) showed no significant difference in either total plaque volume or plaque composition between the two patient groups at baseline.

With CT we diagnosed significant stenoses in 85 segments and CAG diagnosed significant stenoses in 67 segments. Concordance was observed in 59 segments. Stenosis discordance was distributed as follows: CT positive/CAG negative (26 segments): ten segments considered to contain significant stenoses at the CT scans had insignificant stenoses at the CAG and 16 segments considered to have significant stenoses at the CT scans were without stenoses at the CAG; CT negative/CAG positive (eight segments): two stenoses diagnosed as significant at the CAG were in close proximity to a stent and therefore probably missed by CT and one segment containing a significant stenosis at the CAG could not be identified on the corresponding CT scan. Finally, three segments were considered to contain insignificant noncalcified plaques and two segments were considered to contain insignificant mixed plaque on the CT scans.

The radiation dose for the CAG scans ranged between 2 and 10 mSv.

### 3.4. Plaque Development

**Figure 3:**
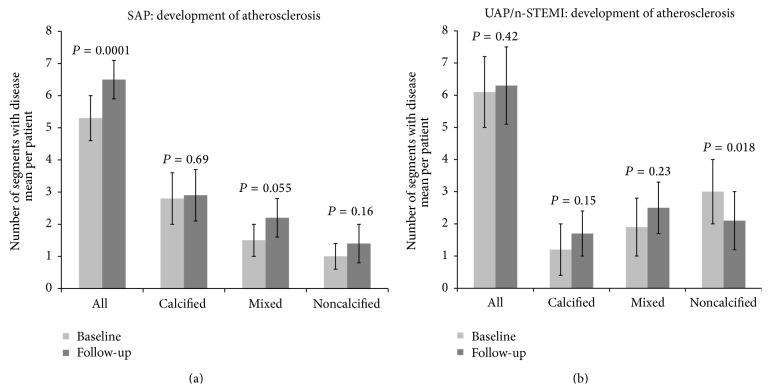
The development of atherosclerosis during one year of follw-up, ((a) stable angina pectoris (SAP) and (b) unstable angina pectoris/non-ST-elevation myocardial infarction (UAP/n-STEMI)). The first two rows display all segments with atherosclerotic plaques; the next six rows display the allocation of the segments into the three atherosclerotic subtypes.

Figure 3(a) shows the development of atherosclerosis in the SAP group, and Figure 3(b) in the UAP/n-STEMI group. After one year we found an increase in the number of segments with disease in the SAP group (5.3 ± 0.71 versus 6.5 ± 0.61, *P* = 0.0001 (*n* = 32)). This was caused by an increase in the number of segments containing mixed plaques (1.5 ± 0.53 versus 2.2 ± 0.62, *P* = 0.055). We detected no difference in the number of segments with disease in the UAP/n-STEMI group after one year (6.1 ± 1.1 and 6.3 ± 1.2, *P* = 0.42 (*n* = 15)). However, in this group we observed marked differences in the subtype of atherosclerosis, with a decrease in the number of segments with noncalcified plaques (3.0 ± 1.0 versus 2.1 ± 0.8, *P* = 0.018). We detected no difference in the number of atherosclerotic segments at baseline between the group of patients not completing the follow-up scan and the group of patients completing follow-up (*P* = 0.27).

### 3.5. Comparison of CT with IVUS and CAG during Follow-Up

The follow-up data achieved by IVUS were in accordance with the follow-up data achieved by CT. After one year IVUS showed a borderline statistically significant increase in the volume (mm^3^) of atherosclerosis in the SAP group (348 ± 71 versus 375 ± 65, *P* = 0.079) and a significant increase in the volume of fibrofatty tissue (24 ± 5 versus 33 ± 11, *P* = 0.031). Consistent with the CT-findings, the IVUS data showed no difference in the volume of atherosclerosis in the UAP/n-STEMI group after one year (413 ± 125 versus 438 ± 125, *P* = 0.28). Furthermore, in the UAP/n-STEMI group we found consistency regarding the change in subtype of atherosclerosis. IVUS detected an increase in the volume of dense calcium (19 ± 9 versus 26 ± 11, *P* = 0.038), which is in accordance with the decrease in noncalcified plaque volume detected by CT.

At follow-up six patients needed additional PCI. Three of these patients were correctly identified as having a significant stenosis at the follow-up CT scan. Out of the remaining three patients, one presented at the re-CAG with a stenosis that could not be identified at the follow-up CT scan because of its in-stent location. In the last two patients, we correctly identified atherosclerotic lesions in the segments undergoing PCI at the re-CAG, but the stenosis was not considered significant at the follow-up CT scan.

## 4. Discussion

While our CT results confirm that patients presenting with UAP/n-STEMI have more noncalcified plaques than patients presenting with SAP, the most important new finding is that vulnerable plaques in UAP/n-STEMI patients appear to stabilize on medical treatment while atherosclerosis in SAP patients appears to be a slowly progressing disorder despite medical treatment. These findings motivate aggressive medical treatment and give a pathoanatomical explanation for the improved outcome in terms of mortality and morbidity after acute coronary syndrome when relevant medication is instituted.

The differences in the plaque composition between patients presenting with SAP and UAP are found in other MDCT-studies [3–5, 7] and further confirmed by Pundziute et al. [6], who also verified the CT-findings by IVUS. However, implications for the progression of the atherosclerotic disease and for the prognosis of the patient remain unknown. Pundziute et al. [14] found that future cardiovascular events were associated with extensive atherosclerosis on MDCT. The same was found by Øvrehus et al. [19] in a group of patients with suspected SAP. Stone et al. [20] have identified plaques with an elevated risk of future cardiovascular events when plaque burden was >70% of the surface area, the minimal luminal area ≤4.0 mm^2^, or when the plaque was characterized as thin cap fibroatheromas by IVUS at baseline. The positive predictive value was low; only 26 of 595 thin cap fibroatheromas identified at baseline were sites for recurrent disease.

None of these studies repeated the CT or IVUS scan in case of an adverse event. Consequently, the composition of the plaque responsible for the new event was not displayed at the time of the event. Follow-up studies with repeated IVUS examinations [21, 22] or MDCT scans [23] in patients without clinical events have demonstrated changes in atherosclerotic plaque composition after initiating lipid lowering therapy in patients with elevated risk of or event documented coronary artery disease. These studies focused on the effect of lipid lowering therapy on plaque burden and demonstrated that intensive lipid lowering therapy reduced the atheroma volume [21, 22] and the noncalcified plaque volume [23]. Brown and Zhao [24] report that IVUS-derived indexes of plaque size failed to predict cardiovascular events.

The invasive nature of IVUS with the associated risks precludes its use in asymptomatic patients and highlights the need for a noninvasive technique [25]. A direct comparison of CT and IVUS data is difficult because different definitions and classification systems are used. The atherosclerotic plaques classified by CT were divided into three groups while the IVUS classification system operates with four different plaque components. Therefore, we never intended to perform a direct comparison between the two imaging modalities but instead sought to demonstrate the same tendency using the different techniques. It can be difficult to obtain IVUS images when patients have severe coronary artery disease. For this reason we were unable to obtain IVUS from three patients and in other patients we were unable to obtain IVUS images from the vessels or vessel segments with most severe atherosclerosis. This may explain the lack of difference at baseline between SAP and UAP/N-STEMI. In the follow-up comparison with the paired data a similar development is, however, documented with both CT and IVUS techniques.

Although only five SAP patients and one UAP/n-STEMI patient needed additional clinically guided PCI at the follow-up examination, cardiac CT revealed significant changes in both subtype and number of segments with atherosclerosis. Albeit only borderline statistically significant, the changes in subtype of atherosclerosis were confirmed by IVUS in our reduced sample size of patients completing follow-up examinations with CAG and IVUS.

The differences in baseline characteristics regarding the cardiovascular risk and prior use of statin therapy might have influenced the plaque development and could indicate that SAP and UAP/n-STEMI are different disease entities. This problem is also dealt with in question regarding rehabilitation and medical therapy.

Even so, our findings suggest that atherosclerosis, particularly in SAP patients, is a progressive disease despite prophylactic medical treatment. The aggressive medical treatment instituted in the UAP/n-STEMI patients may explain the lack of disease progression in this group. Alternatively, a longer follow-up time might have revealed a slower progression or even progression after the first year.

We only divided the atherosclerotic segments into noncalcified, mixed, and calcified subtypes. More thorough analysis of the individual plaques and further subdivision of the noncalcified plaques into lipid-rich and fibrotic subtypes might be preferable to optimize identification of the vulnerable plaques and predict future ischemic events, because not all noncalcified atherosclerotic plaques are equally rupture prone [1]. However, such an approach introduces other limitations as CT density values in lipid-rich and fibrotic plaques are overlapping [26, 27] and inconsistent [13], suggesting that the current CT technology cannot perform reliable distinction between lipid-rich and fibrotic plaques [25].

The proportion of coronary artery segments containing atherosclerosis was rather high in the present study. This was expected, as we only included patients referred to invasive CAG due to cardiovascular symptoms. Hence, the atherosclerotic process was already advanced challenging not only the IVUS examinations but also the registration of differences of progression and characterization of atherosclerotic segments at follow-up. Even so, we demonstrated a significant difference in both the number and subtype of atherosclerotic segments at baseline between the two patient groups and a difference in the number and composition of the atherosclerotic segments after one year.

Because coronary artery disease was rather advanced in our study cohort, several patients were referred to coronary bypass operation and excluded from the follow-up examinations. We excluded patients referred to CABG for two reasons: first, we considered the follow-up examination with both CAG and IVUS to be more complex and secondly the native vessels often undergo extensive atherosclerotic changes after a coronary bypass operation, which could interfere with our results. Because the proportion of patients, who did not complete follow-up, did not differ between our study groups, we do not suspect any bias caused by differences in disease entity.

We only reported the number of segments with atherosclerosis rather than the total plaque burden. We chose this approach for several reasons. Until now no consensus to quantify total plaque burden is established. Available software cannot reliably quantify total plaque burden and manual estimation is subjected to inter- and intraobserver variability. This might mask our results during the follow-up because an increase in plaque burden within already affected segments might not be registered.

This study, although illustrating a development in the amount and subtype of atherosclerosis over time, cannot clarify the natural history of atherosclerosis because all patients received prophylactic medical treatment after inclusion. Consequently, our study reports the course of atherosclerosis under medical treatment.

## 5. Conclusion

Cardiac CT and IVUS consistently reveal that the extension and subtype of atherosclerosis differ and progression varies between patients with SAP and UAP/n-STEMI. Despite medical treatment and other interventions like smoking cessation, and physical rehabilitation, atherosclerosis appears to progress in patients with SAP, whereas the same interventions seem to stabilize the atherosclerotic lesions in patients with UAP/n-STEMI.

## Figures and Tables

**Table 1:** Patient characteristics at baseline.

Patient characteristics	SAP *n* = 40	UAP/n-STEMI *n* = 20	*P* value
Male gender *n* (%)	35 (87.5%)	15 (75%)	0.28
Age – year (±95% CI)	63 (±2.5)	59.7 (±4.3)	0.17
Ejection fraction (±95% CI)	59.4 (±2.6)	55.3 (±3)	0.056
BMI (±95% CI)	27.6 (±1.1)	27.6 (±1.6)	0.98
Treatment for dyslipidemia *n* (%)	34 (85%)	5 (25%)	<0.0001
Treatment for hypertension *n* (%)	28 (70%)	7 (35%)	0.01
Family history of IHD *n* (%)	20 (50%)	4 (20%)	0.048
Type 2 diabetes mellitus *n* (%)	0 (0%)	3 (15%)	0.033
Smoking			0.57
No *n* (%)	11 (27.5%)	4 (20%)	
Active *n* (%)	10 (25%)	8 (40%)	
Former *n* (%)	19 (47.5%)	8 (40%)	

**Table 2:** Risk factor management at follow-up.

Risk factor management at follow-up	SAP *n* = 32	UAP/n-STEMI *n* = 15	*P* value
Aspirin *n* (%)	31 (97%)	15 (100%)	1
Clopidogrel *n* (%)	19 (59%)	14 (93%)	0.02
Statins *n* (%)	28 (88%)	15 (100%)	0.29
Beta-blockers *n* (%)	19 (59%)	9 (60%)	0.97
ACE-inhibitors/AT2-antagonists *n* (%)	15 (49%)	8 (53%)	0.68
Smoking			0.60
No *n* (%)	7 (22%)	3 (20%)	
Active *n* (%)	6 (19%)	1 (7%)	
Former *n* (%)	19 (59%)	11 (73%)	

**Table 3:** IVUS data at baseline.

IVUS data baseline	SAP *n* = 29	UAP/n-STEMI *n* = 19	*P* value
Total plaque volume mm^3^ mean (±95% CI)	339 (±56)	392 (±72)	0.23
Fibrous plaque volume mm^3^ mean (±95% CI)	109 (±23)	131 (±37)	0.27
Fibro fatty plaque volume mm^3^ (±95% CI)	22 (±5)	27 (±9)	0.26
Necrotic core plaque volume mm^3^ (±95% CI)	47 (±13)	50 (±15)	0.74
Dense calcium plaque volume mm^3^ (±95% CI)	22 (±7)	23 (±11)	0.83

## Abbreviations

BMI: Body mass index
CAG: Coronary angiography
CCTA: Coronary computed tomography angiography
CI: Confidence interval
CT: Computed tomography
IVUS: Intravascular ultrasound
MDCT: Multidetector computed tomography
n-STEMI: Non-ST-elevation myocardial infarction
PCI: Percutaneous coronary intervention
SAP: Stable angina pectoris
UAP: Unstable angina pectoris.
